# Study of helical flow inducers with different thread pitches and diameters in vena cava

**DOI:** 10.1371/journal.pone.0190609

**Published:** 2018-01-03

**Authors:** Ying Chen, Xiaoyan Deng, Xinying Shan, Yubin Xing

**Affiliations:** 1 Key Laboratory for Biomechanics and Mechanobiology of Ministry of Education, School of Biological Science and Medical Engineering, Beihang University, Beijing, China; 2 Beijing Advanced Innovation Centre for Biomedical Engineering, Beihang University, Beijing, China; 3 Department of Infection Management and Disease Control, The General Hospital of People’s Liberation Army, Beijing, China; University at Buffalo - The State University of New York, UNITED STATES

## Abstract

Pulmonary embolism is a severe, potentially life-threatening condition. Inferior vena cava filters have been used to prevent recurrent pulmonary embolisms. However, the build-up of thrombosis in vena cava filters after deployment presents a severe problem to patients. Previous studies proposed that filters with helical flow are beneficial and capable of alleviating this problem. In this study, the hemodynamic performances of four typical helical flow inducers in the vena cava are determined using computational fluid dynamics simulations (steady-state and pulsatile flow) and compared. Pilot in vitro experiments were also conducted. The simulation results demonstrate that large-diameter inducers produce helical flow. Among inducers with identical diameter, those with a smaller thread pitch are more likely to induce increased helical flow. We also observed that the small thread pitch inducers can yield higher shear rates. Furthermore, a large diameter, small thread pitch helical flow inducer increases the time-averaged wall shear stress and reduces the oscillating shear index and relative residence time on the vessel wall in the vicinity of the helical flow inducer. In vitro experiments also verify that large diameter inducers generate a helical flow. A notable observation of this study is that the diameter is the key parameter that affects the induction of a helical flow. This study will likely provide important guidance for the design of interventional treatments and the deployment of filters associated with helical flow in the vena cava.

## Introduction

**Fig 1:**
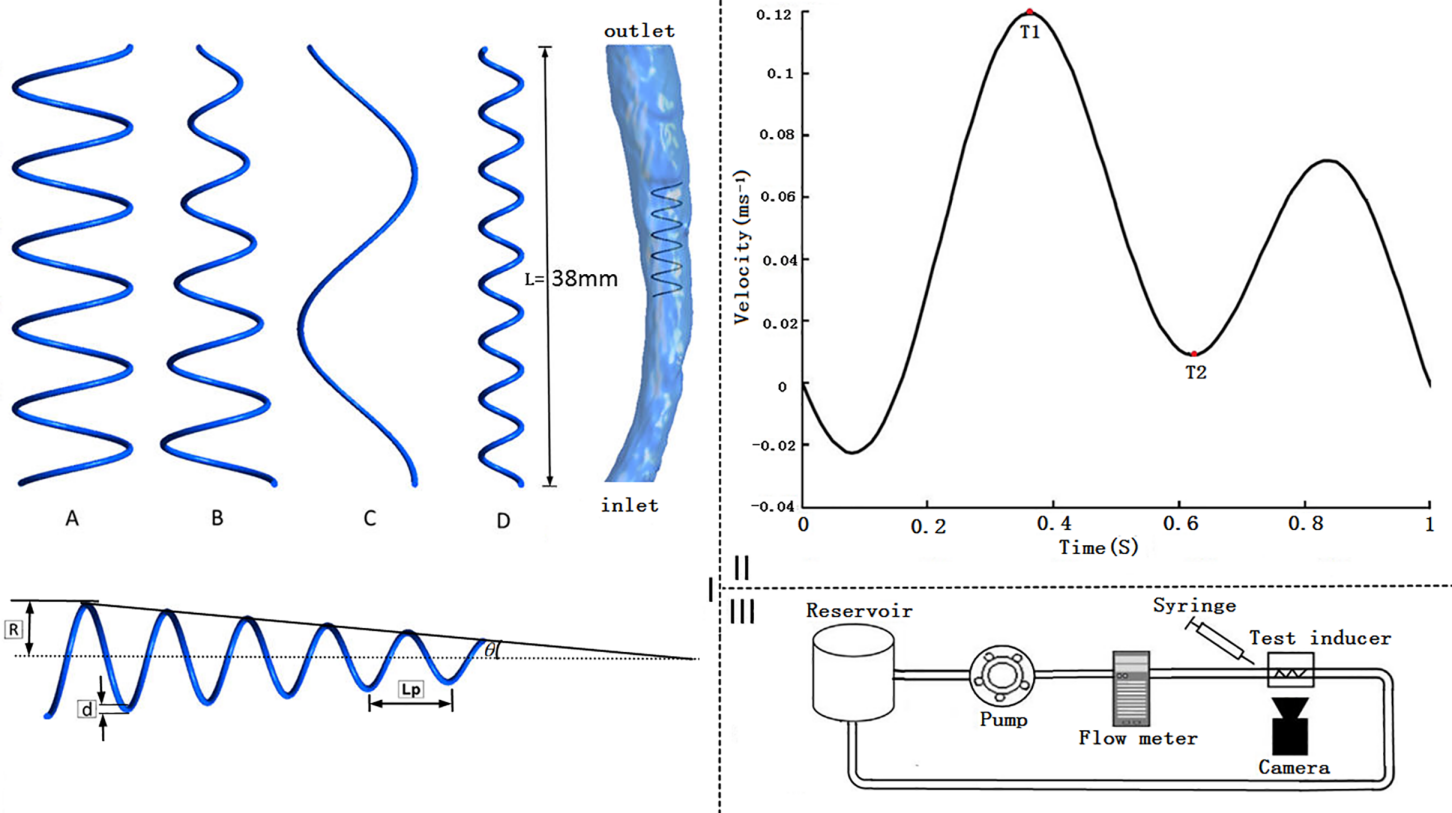
Helical flow inducers, vena cava model, inlet waveform velocity, and experimental set-up scheme. I: Schematic of the four helical flow inducers (A, B, C, and D) using the different thread pitches and diameters considered herein. The figure also shows an inducer in the vena cava used for simulation and a diagram of depicting the geometry parameters. II: Inlet inferior vena cava velocity waveform velocity used in the pulsatile flow computations. III: Schematic of the experimental set-up.

Inferior vena cava (IVC) filters have been utilized in the prevention of pulmonary embolism (PE) when anticoagulation is contraindicated in patients with proximal deep vein thrombosis (DVT) [1]. Although vena cava filters (VCF) have been extensively used clinically, the filter’s re-blockage problem after deployment remains unsolved [2]. Helical flow has been introduced to improve the hemodynamic performance of vascular devices such as arterial grafts and stents. Morbiducci investigated the physiological relevance of helical flow in the aorta and asserted that it could optimize the fluid transport process in the cardiovascular system [3]. Moreover, their study indicated that helical flow played a positive role by preventing the bypassed arteries from developing intimal hyperplasia [4]. Caro and Zheng demonstrated that helical-shaped bypass grafts significantly reduced thrombosis and intimal hyperplasia compared to conventional shunts [5, 6]. Hajin and colleagues proposed a fluid-based optimal design of a helical vascular graft for disturbed stenotic flow [7]. Wen et al. numerically simulated the hemodynamic characteristics of several arteriovenous grafts with various helical shapes, and their simulation results showed the helical grafts suppress the disrupted shear stress distribution in the venous segment compared to the conventional straight graft [8]. Our previous study also demonstrated that use of a novel helical flow vena cava filter design could result in hemodynamic performance improvements in the vena cava [9, 10]. The induction of helical flow is likely to minimize clinical risks of deep venous thrombosis and pulmonary embolisms. Although extensive research has supported the beneficial effects of helical flow, minimal work has been conducted to investigate the mechanism by which helical flow inducers induce helical flow in a vena cava system. In Fig 1I, we observe that helical flow inducers are mainly characterized by parameters such as the thread pitch (Lp), diameter (2R), and conical angle (*θ*). This study investigates the types of helical flow inducers that can produce a more significant helical flow in the vena cava. The hemodynamic performances of four typical helical flow inducers are compared by performing computational simulations and conducting pilot in vitro experiments.

## Materials and methods

### Simulation model and geometry description

The anatomic vena cava model in association with the use of computed tomography (CT) images from volunteers was approved by the Ethical Committee of the PLA General Hospital and was carried out in accordance to hospital regulations.

In this study, four typical designs of a helical flow inducer were compared (Fig 1I), based on the use of a Greenfield filter [11, 12], its employment in the vena cava, and extraction of relevant parameters from the literature [13]. The following models are considered: model A, in which the inducer has a large and an invariable diameter of 10 mm, and a thread pitch of 7 mm; model B, in which the inducer has a conical angle with a diameter that varied from 10 mm to 3.5 mm in the blood flow direction, and a thread pitch of 7 mm; model C, in which a helical flow inducer has a large and invariable diameter of 10 mm with a thread pitch of 27 mm; and model D, in which the helical flow inducer has an invariable small diameter of 3.5 mm, and a thread pitch of 5 mm. Compared to the previous, novel helical flow vena cava filter, and the Greenfield filter prototype [9, 11], in these four inducers, the circular cross-section diameter of the inducer leg is 0.6 mm, and the total length of each inducer (L) is 38 mm. In the present study, we focus on the hemodynamic performance of the helical flow inducers in the vena cava.

All the inducer models were created with different thread pitches and diameters. The inducers have specific geometric parameters. The helix curvature (k) and torsion (*τ*) are described as follows [14],

$$k=\frac{R}{R^{2}+\beta^{2}}$$ (1)

$$\tau =\frac{\beta }{R^{2}+\beta^{2}}$$ (2)

where *β* is defined as

$$\beta =\frac{\mathrm{Lp}}{2\pi }$$ (3)

The number of helical turns is described in accordance to Eq 4:

$$n=\frac{L}{\mathrm{Lp}}$$ (4)

where n represents the helix number of the inducer helices. *β* represents the numerical index of the inducer’s thread pitch, and k and *τ* represents the parameters expressing the relationships between the number of thread pitch and radius, respectively.

Specific geometric parameters of the inducer models are listed in Table 1. All of the inducer models were created using the computer-aided design software, Pro/Engineer Wildfire 4.0 (Parametric Technology Corporation, Needham, MA, USA). The vena cava model was reconstructed based on computed tomography (CT) scan images. These images were used to reconstruct the model using the Mimics software (Version 9.0, Materialise, Ann Arbor, MI, USA). Standard image processing analyses were applied to the models using Geomagic Studio (Version 12, Raindrop, USA) followed by further processing using Pro/Engineer Wildfire 4.0 (Fig 1I). Control simulations of the vena cava without a helical flow inducer, referred to as model E, and were also conducted for comparison purposes.

**Table 1:** Geometric parameters for each studied case. Case A_1_ represents the inducer of the helical filter of the previous study [9].

Case	A	B	C	D	A_1_
R(mm)	5	1.75–5	5	1.75	1.5–9
Lp(mm)	7	7	27	5	5
*β*(mm)	1.14	1.14	4.30	0.80	0.80
n	5.43	5.43	1.41	7.60	7
k(1/m)	190.1	401.02–190.10	115.04	473.52	520.25–110.25
*τ*(1/m)	43.37	261.42–43.37	98.86	215.32	276.0–9.75

### Assumptions

Perktold has indicated in his study that similar differences in the flow features of non-Newtonian and Newtonian numerical simulations are elicited in a bifurcation in an aneurysm [15]. However, it is unknown on whether the same findings apply in the case of the vena cava. Therefore, in the present study Newtonian and non-Newtonian flow simulations were performed for steady flows. Furthermore, the vena cava vessel wall was assumed to be rigid and non-slipping in both the steady flow and pulsatile flow cases. The flow through the inferior vena cava (IVC) is characterized by the Reynolds number Re = *ρ*UD/*μ*, where *ρ*, U, D, and *μ* are the density, velocity of the flow, diameter of the IVC, and dynamic viscosity, respectively. The Reynolds numbers of blood in the IVC is approximately equal to 600, or lower [16–18]. Therefore, the simulations were performed by assuming laminar flow conditions.

### Governing equations

The simulations for flow motion were based on the three-dimensional incompressible Navier–Stokes equations [19]:

ρ((∂υ/∂t)+(υ⋅∇)υ)=−∇p+∇⋅τ (5)

∇⋅υ=0 (6)

where *υ* and *p* respectively represent the fluid velocity vector and the pressure. The density of blood was taken as ρ = 1050 kg/m^3^, and the blood tension tensor *τ* was

$$\tau =2\eta (\dot{\gamma })T$$ (7)

where T and $\dot{\gamma }$ respectively represent the deformation rate tensor and shear rate, and *η* is the blood viscosity that is a function of the shear rate.

For the non-Newtonian blood flow simulations, we choose the Carreau model for obtaining the blood viscosity, expressed by the following equation:

$$\eta (\dot{\gamma })=\eta_{\infty }+(\eta_{0}-\eta_{\infty }){\left[1+{\left(\lambda \dot{\gamma }\right)}^{2}\right]}^{\left((n-1)/2\right)}$$ (8)

where *η*_∞_ = 3.45×10^-3^kg/ (m s), *η*_0_ = 5.6×10^-2^kg/ (m s), n = 0.3568, and *λ* = 3.313 s [20].

Herein, it should be mentioned that there are also many other non-Newtonian viscosity models, such as the Bingham fluid and the Casson models. In the vena cava system, it is still unknown how the adoption of different non-Newtonian models could influence simulation results.

For the Newtonian blood flow simulation, blood viscosity was regarded as a constant, whereby, *η*_∞_ = 3.45 × 10^−3^ kg/ (m s).

### Boundary conditions and mesh generation

The boundary conditions for the steady flow simulations were as follows. Inlet: The inlet velocity is 0.1 m/s. Outlet: The outlet was set to be an outflow, representing a completely developed flow.

The boundary conditions for the pulsatile flow simulations were as follows:

Inlet: The blood flow in the inferior vena cava was influenced by the contraction of the heart. The IVC exhibited pulsatile waveforms with reverse flow, with two peaks occurring in each cardiac cycle. Considering the Doppler blood flow waveforms in the IVC reported in the study of Zhang [21], the flow waveforms are approximated using the smooth periodic function plotted in Fig 1II. Therefore, the time-dependent parabolic flow velocity waveform shown in Fig 1II is set at the inlet.

Outlet: The boundary conditions were similar to those set for the steady flow computation, and were was also set to be the outflow boundary conditions.

The finite volume method was used for the simulations. ICEM (ANSYS Inc., Canonsburg, PA, USA) was used to post-process the simulation data. All the computational models were meshed with tetrahedral and hexahedral cells using ICEM. The ANSYS Fluent solver (ANSYS Inc., Canonsburg, PA, USA) was being used for these simulations. In order to ensure that the results were mesh-independent, the grid-adaptation technique was used, which refined the grid based on the geometric data and numerical solution. Boundary layers in the vicinity of the vessel wall were prescribed as follows: the total height was set to 0.2 mm, the height ratio was set to 1.2, and the number of the boundary layers was set to 3. Finally, the numbers of mesh elements of scenarios A, B, C, D, and E (without helical flow inducers) have been listed in the Table 2. In each control volume element, the pressure and momentum were discretized according to a second-order scheme. The iterative process in the computation was terminated when the residual continuity and velocity values were all below the convergence criterion that was set at 1.0 × 10^−5^. In the pulsatile flow simulations, six cycles were required, with 200 steps in each cycle, to obtain convergence for the transient analysis. Pulsatile calculations were conducted on a computer equipped with a 2.40 GHz Intel(R) Xeon(R) Central Processing Unit (CPU) processor and 64GB RAM. The computational time-span approach a week for each scenario.

**Table 2:** Number of elements used for the various cases studied.

Case	A	B	C	D	E
Element numbers	2,698,791	2,304,422	1,967,839	2,209,766	850,889

### Hemodynamic indicators

To characterize the helical flow, the area-weighted average of the helicity density H_d_ was calculated, as defined by Eq 9 [22]:

$$H_{d}=\upsilon \cdot (\nabla \times \upsilon )=\upsilon \cdot \omega$$ (9)

$$H_{\left(\mathrm{average}\right)}=\left(\int_{s}H_{d}ds\right)/s$$ (10)

where *ω* = ∇ × *υ* is the vorticity field of the flow, and *S* is the cross-sectional area.

Hemodynamic parameters were evaluated based on the shear stress on the vessel wall throughout the cardiac cycle, including the time-averaged wall shear stress (TAWSS), oscillating shear index (OSI), and relative residence time (RRT). TAWSS was calculated in accordance to the following equation:

$$TAWSS=\frac{1}{T}\int_{0}^{T}|WSS(s,t)|dt$$ (11)

where T is the cardiac cycle period, WSS is the instantaneous wall shear stress vector, and s is the position on the vessel wall.

Ojha had previously reported that the pathogenesis of intimal hyperplasia of the vessel wall is correlated with low WSS and high OSI values [23, 24]. The WSS vector with a high frequency change in direction had a high OSI value. OSI is defined as [25],

$$\mathrm{OSI}=\frac{1}{2}\left[1-\left(\frac{\left|\frac{1}{T}\int_{0}^{T}\mathrm{WSS}\left(s,t\right)\cdot \mathrm{dt}\right|}{\frac{1}{T}\int_{0}^{T}\left|\mathrm{WSS}\left(s,t\right)\right|\mathrm{dt}}\right)\right]\;0\leq \mathrm{OSI}\leq \frac{1}{2}$$ (12)

OSI indicates the regions where the endothelial shear stress changes between positive and negative values during the cardiac cycle.

The relative residence time (RRT) was also calculated in accordance to the equation:

$$\mathrm{RRT}=\frac{1}{\left(1-2\cdot \mathrm{OSI}\right)\cdot \mathrm{TAWSS}}$$ (13)

RRT is a useful hemodynamic parameter of the shear environment that combines TAWSS and OSI, and it reflects the residence time of flow particles near the vessel wall.

### Simulation results

#### Helicity

**Fig 2:**
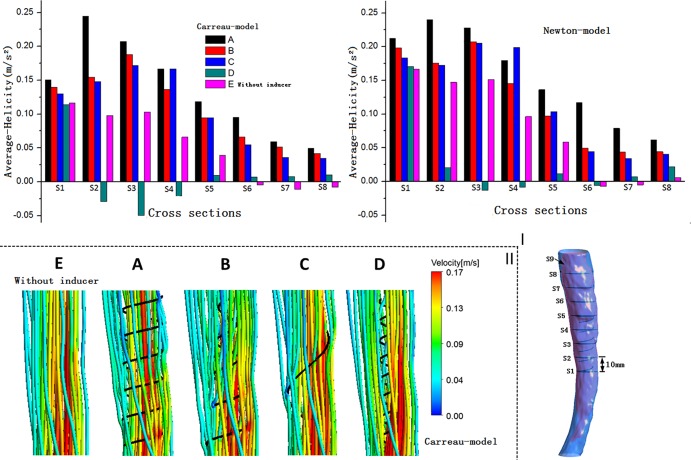
Helicity and streamlines. I: Eight representative slices in the vena cava model, with adjacent slices spaced 10 mm apart; plots of area-weighted averages from steady flow computations of helicity (H_d_) for all the eight slices of for each case. The left panel shows the elicited results using the Carreau model, while the right panel presents the results elicited using the Newton model. II: Velocity streamlines near the inducers are obtained from the steady flow computations.

Fig 2I shows eight representative slices (S1–S8) in the vena cava model that are selected for the study of each model. The resulting area-weighted averages of H_d_ are plotted for S1–S8 under a steady flow conditions. Fig 2I suggests that a large-diameter inducer (models A and C) induces a higher H_d_ than a small-diameter inducer (model D) does. Comparison of models A and C in Fig 2I also indicate that among inducers with identical diameters, the pitch inducer with a smaller thread yields a marginally higher H_d_. In addition, comparing models A and model B, it can be deduced that the conical angle of an inducer is likely to marginally reduced H_d_. We observe in the figure that model E, which does not contain a helical flow inducer, also exhibits a none-zero H_d_, that may be attributed to the curvature of the vena cava. Model D has a very small diameter, and H_d_ value that is lower than that of model E. The reason that model D exhibits lower helicity than model E is that an inducer with a very small diameter is likely to cause flow rotation both in the clockwise and counterclockwise directions (because certain sides exhibit negative helicity). With regard to H_d_, there are similarities between the Newton model simulation and the Carreau model simulations.

### Streamlines

Velocity streamlines from steady-state simulations near the helical flow inducer in the four models are shown in Fig 2II, with color coding for the velocity magnitude (speed). For comparison, the model without a helical flow inducer is also depicted. In model A, helical flow is more evident, while in model D the streamlines are smoother. In addition, the flow velocity is in general higher at the inducer’s center in models A and B. Because the flow streamlines obtained using the Newton and Carreau models did not differ significantly, only the streamlines from the Carreau model are shown in the Fig 2II.

### Shear rate

**Fig 3:**
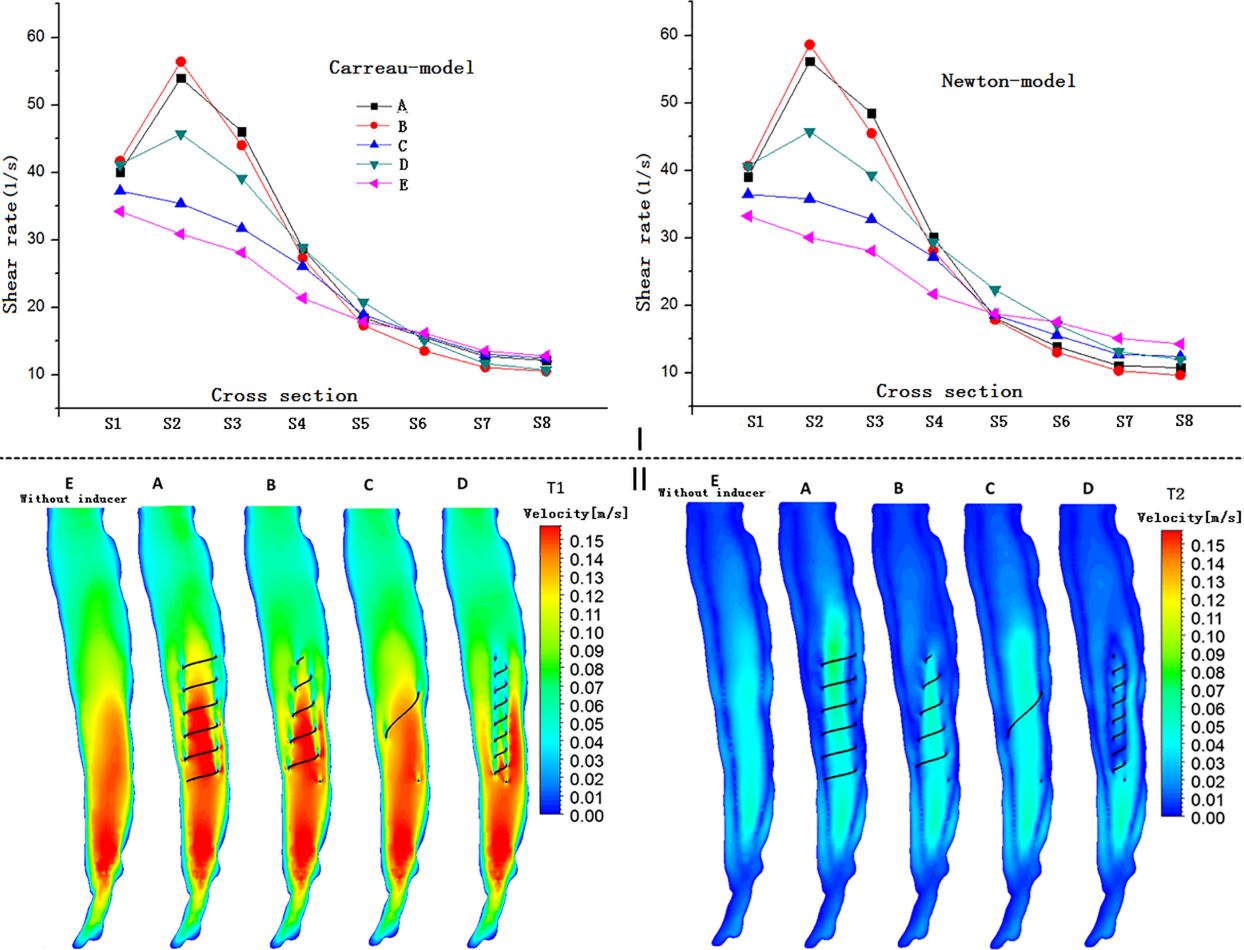
Shear rates and velocity distributions for the five modes. I: Shear rates for the eight representative slices of the vena cava using the Carreau and Newton models in the steady flow computations. II: Longitudinal slice (Fig 2I, S9) velocity distributions for T1 and T2 based on pulsatile flow computations.

Fig 3I illustrates the shear rate profiles for the eight representative cross-sections of the vena cava using the different models. Fig 3I illustrates that in all the models, the shear rate of the blood flow adjacent to the inducers is much higher relative to other regions within the vena cava. Near the inducer, models A and B evidently induce progressively higher shear rates at spatial locations ranging from S1 to S4, with the maximum shear rate value elicited by models A and B in S2. In comparison, the shear rates for models C and D are lower. However, Model D yields a higher shear rate than model C. In S5–S8, there is no evident difference between the different models A–E. These results also suggest that all inducers generate higher shear rates compared to the case where no helical flow inducer was used. In terms of the shear rates also, there are similarities between the Newton simulation and Carreau model simulations.

### Flow patterns

Fig 3II shows the velocity alteration in the lengthways side (S9) in each case during the cardiac cycle, particularly at T1 and T2. In general, except for the case where the helical flow inducer was placed inside, each of the helical inducers relevant to the studied cases did not significantly change the velocity of the blood flow within the vena cava at any instant during the cardiac cycle. However, for models A and B, the increase of the flow velocity in the center of the inducer center is more pronounced after the placement of the helical flow inducer both at T1 and at T2. For instance, at peak systole (time = T1), as is evident from the Fig 3II, the central velocity of model A reaches values as high as 0.15 m/s. However, for the model C, the velocity is not evidently affected by the inducer. For the model D, the velocity is actually decreased in the vicinity of the inducer. In conclusion, a large diameter and a pitch inducer with a small thread induce a higher velocity in the center of the inducer.

### Time-averaged wall shear stress

**Fig 4:**
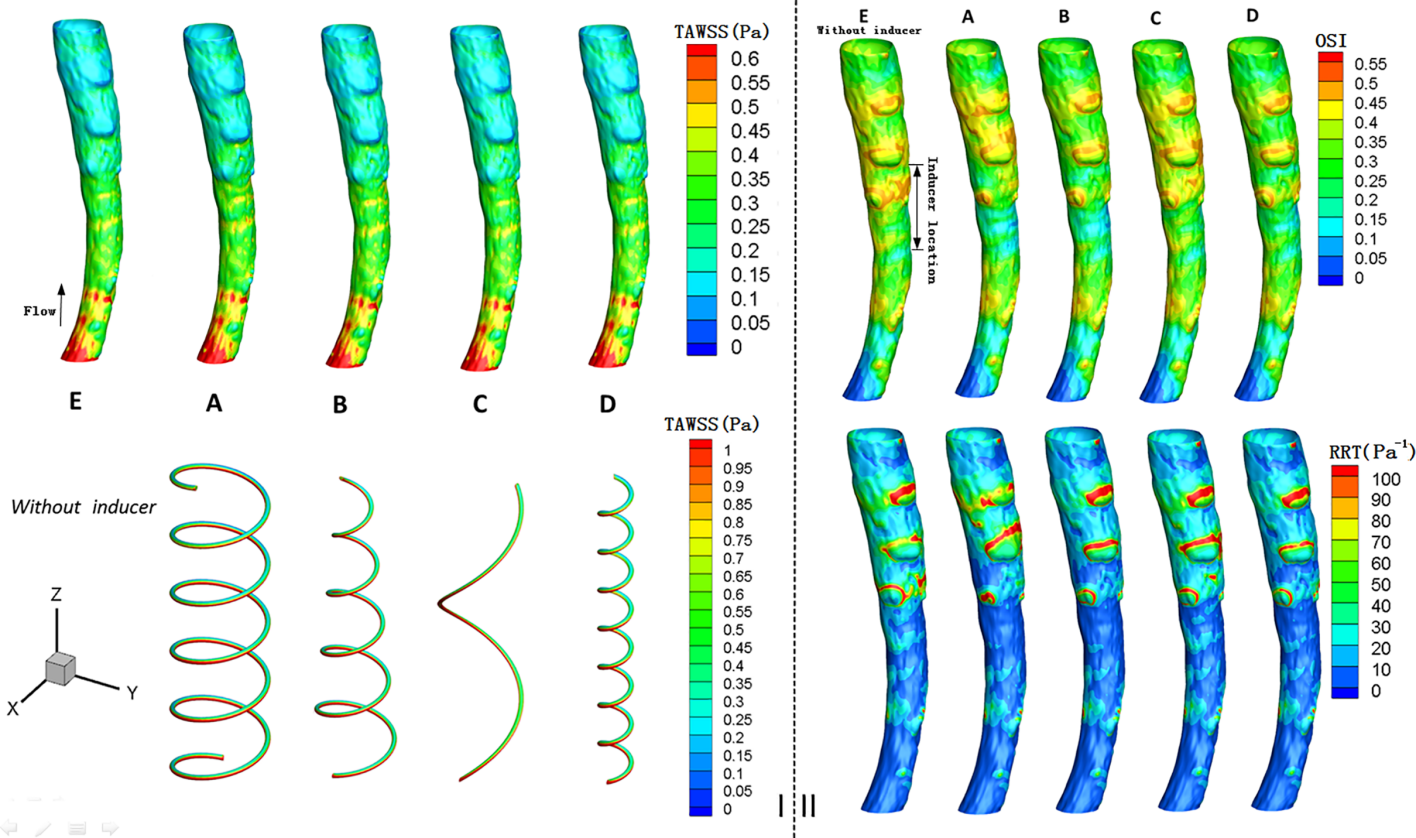
Contours of time-averaged wall shear stress (TAWSS), oscillating shear index (OSI) and relative residence time (RRT), based on pulsatile flow computations. I: Contours of TAWSS (unit: Pa) on the caval wall and inducer. II: Contours of OSI and RRT (unit: Pa^-1^) for the caval wall.

Flow and WSS associated with a hemodynamic medical device are important as they can aid in the prediction of the onset of outcomes such as hemolysis and thrombosis. Low stress levels are generally associated with flow stasis and thrombosis [26]. Fig 4I shows the distributions of the TAWSS on the vessel walls and on the inducers, and the results indicate that the distribution of the TAWSS did not vary significantly across the different models. In particular, for model A, the TAWSS on the vessel wall appears to be slightly higher. For example, in the “Inducer location,” the value of TAWSS on the vessel wall in the vicinity of the inducer in model A, attains a peak value of approximately 0.5 Pa in the center, whereas the peak value of TAWSS in model E is approximately 0.47 Pa. Furthermore, Fig 4I also demonstrates that in each case, the TAWSS values on the inducers that are inside is higher those on the outside.

### Oscillatory shear index and relative residence time

From the Fig 4II, we observe that after the deployment of these four helical flow inducers, the OSI of the vessel wall mainly decreases in the vicinity of the inducer; however, the OSI is not evidently reduced in the downstream. For example, comparison of models E and A at the “Inducer location,” showed that the OSI in the vicinity of the helical flow inducer was clearly decreased from 0.45 to 0.3.

As is evident from Fig 4II, a helical flow inducer decreases the RRT on the vessel wall, particularly for the models A, B and E. Near the helical flow inducer, the effect of the inducer in reducing the RRT is more apparent. Compared with the model E that has no inducer, the value of the RRT on the vessel wall is decreased from 40 Pa^-1^ to 20 Pa^-1^, for model A. Based on the combined analysis of the OSI and RRT, we can deduce that low values of the RRT usually correspond to low values of the OSI, and vice versa.

### In vitro experiments

#### Experimental set-up

**Fig 5:**
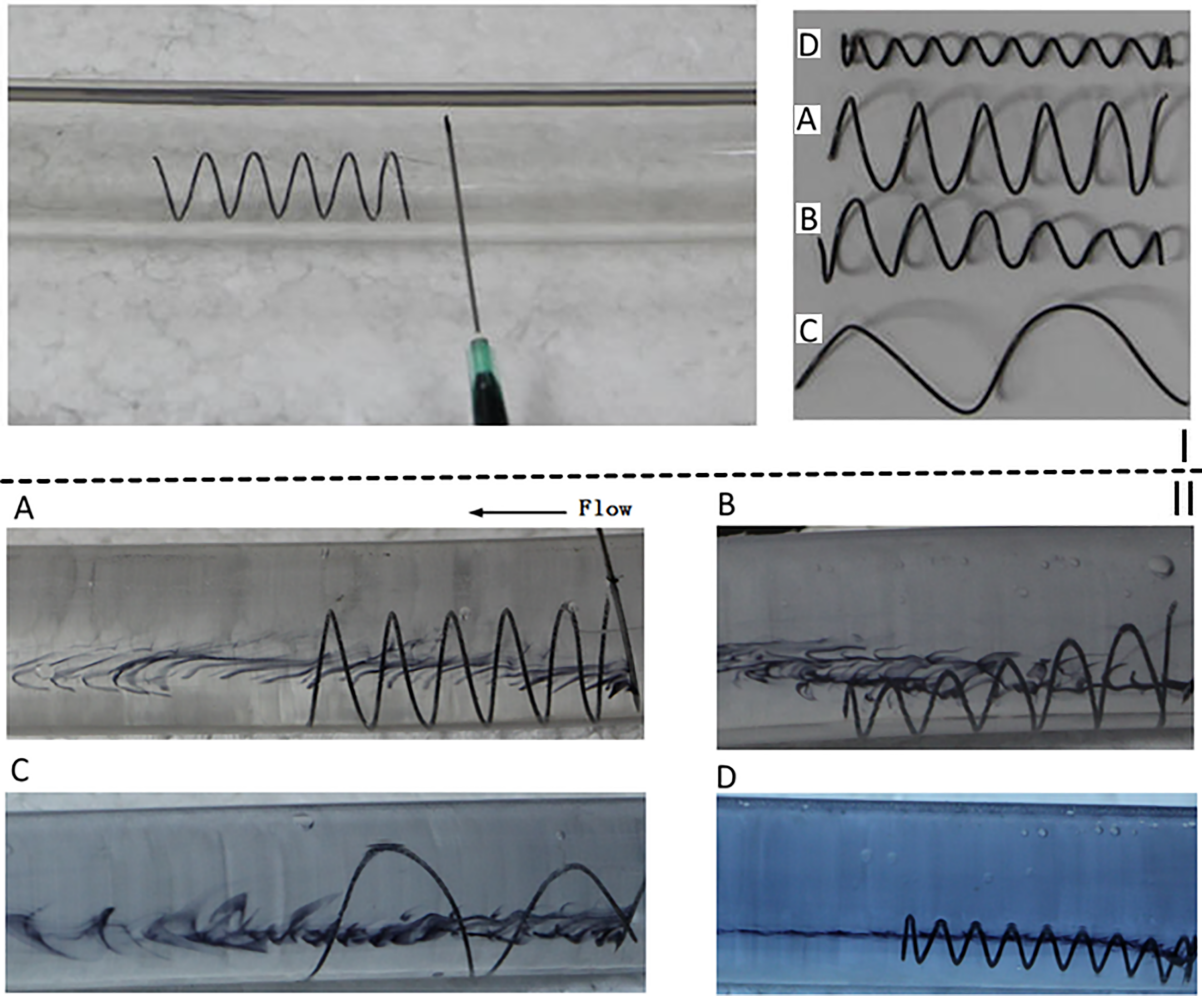
Velocity streamlines for the four cases of in vitro experiments. I: Four types of helical flow inducers used in the in vitro experiments. II: Velocity streamlines corresponding to the four cases of the in vitro experiments.

We adopted the same methods as previously reported, whereby a circulation perfusion system was constructed [9]. A flexible and transparent plastic hose with an inner diameter of 19 mm was used for simulating the vena cava. A flow meter was used to measure the flow velocity. Each inducer was placed in the plastic hose, and a black dye was injected upstream. A digital camera was used to acquire photographs for analyzing the flow patterns. A mixed fluid that mimicked blood comprising 1/3 by volume of glycerol in water was used. Four typical helical flow inducers, corresponding to the numerical simulations described above, were tested. All of the studied inducers (Fig 5I) were constructed using spring processing technology, which uses heat treatment to wind a metal wire. The circular cross-sectional diameters of all the inducers were 0.6 mm, and their lengths were 38 mm. The inducers were fixed using a cotton thread in the upstream. All these experiments were performed under a flow rate of 1L/min [27].

#### In vitro experimental results

Fig 5 shows the photographs of the four scenarios studied based on in vitro experiments. Note that the flow direction in these photographs is from the right to the left. The dye is injected at the right end. Although the mixed liquid generates some bubbles, Fig 5 clearly shows that helical flows are induced in all the cases. In particular, inducer models A and C clearly yielded large helical flow vortices. Model B may induce helical flows in the downstream direction. In the case of model D, several small helical flow vortices were generated inside the inducer. In general, large-diameter inducers generated strong helical flows. The small-diameter inducers mainly generated several eddies insider the inducers. Furthermore, compared with the pitch inducer with a large thread, the pitch inducer small thread induced more vortices in its inner parts. For models A, B, and C, the flows developed larger vortices downstream, while for model D there were no evident vortices in the hose except for those in the inner parts of the inducer. Therefore, from the in vitro experiments, we concluded that the diameter is the key parameter for inducing helical flows.

## Discussion and conclusion

Recently, several studies [28–30] have demonstrated the beneficial effects of helical flows in arterial bypass surgery, arterial stenting, and in the venous system. Intentionally induction of a helical flow in a vena cava vessel is likely to assist in overcoming the challenges of acute DVT and PE. However, with regard to the vena cava system, there is still no clear explanation about how helical flow could prevent thrombus build-up in the filters. The current understanding of the mechanisms regulating the induction of a helical flow in the vena cava is inadequate. In this study, we conducted numerical simulations of the blood flow using four typical helical flow inducers, and analyzed different biomarkers and hemodynamics parameters, such as the helicity density, and the oscillatory shear index of the flow. In addition, we performed in vitro experiments to investigate the streamlines induced by these four helical flow inducers.

This study determined that large diameter inducers are likely to induce stronger helical flows, while pitch inducer with small threads are likely to yield higher shear rates in the vicinity of the inducer. The shear rate values in these cases were less than 60 s^-1^. It is reported that very high shear rates (>10,000 s^-1^) were linked with platelet activation and aggregation, which impels thrombus growth [31]. In the vena cava, the value of the shear rate is significantly smaller than 10 000 s^-1^. Therefore, it may be concluded that the hemodynamic effects between these cases are likely to be marginal in terms of the shear rate.

Comparison of the numerical simulation results from these cases demonstrated that the helical flow inducer with a large diameter and a small thread pitch, induced higher helicity, and reduced the OSI and RRT values. Several studies have demonstrated that high OSI and RRT values are associated with the occurrence of a stent thrombus [32]. Furthermore, among the inducers with identical diameters, the pitch inducer with a large thread could induce lower shear rates in its vicinity. With respect to geometric parameters, lower helix curvature (k) and torsion (*τ*) induce in a straightforward manner increased helical flows. In vitro experiments also verified that the helical flow inducer with a large diameter could induce increased helical flows.

Apart from the thread pitch and diameter, helical flow inducers are also characterized by the conical angle, which indicates that the helical flow inducer has a variable diameter. Numerous filters such as the TrapEase and Mobin–Uddin filter, which have a conically shaped structure, exhibit unfavorable hemodynamics; this is because this shape results in clot lodging and stagnation along the vessel wall, which will likely contribute to the formation of additional clots [26, 33]. Therefore, in general, the inducer with a large diameter, a large thread pitch, and without a conical angle, exhibits a better hemodynamic performance. In this study, we mainly focused on the hemodynamic performance of the helical flow inducers disregarding the manner in which they are fixed to the vessel wall. In consideration of the filter design, these helical flow inducers could be used in tandem with a cone-shaped filter, such as the Greenfield filter and Gunther Tulip filters. For example, model A or B inducers are likely to be suitable for use in tandem with the Greenfield filter. In addition, the results revealed similar flow features between the Carreau model and Newtonian models in numerical simulations.

Certain limitations exist in relation to the in vitro experimental studies conducted herein. Firstly, dye injection provides only qualitative data regarding the fluid flow field. In future work, in order to obtain quantitative data on the fluid velocities and information on the local shear stresses in the fluid flow field, we may consider the use of a different approach such as laser Doppler anemometry, particle image velocimetry, or particle tracking velocimetry. Furthermore, data on the local fluid flow field would provide information on the presence or absence of unfavorable fluid flow patterns that could potentially induce the formation of thrombi. Second, the use of a peristaltic pump is non-physiological since flow in the vena cava is considered to be largely continuous, with variations based on the phasic respiration changes in the right atrium. The conditions used herein are likely to be more or less different from in vivo conditions.

In conclusion, the reported study is only a preliminary study. The performance characteristics of the helical flow inducers were determined primarily based on computational simulations. To validate the conclusions of the study, animal experiments or human clinical trials should be performed in the future.

## Supporting information

S1 Table **A-D.** Original data of helicity and shear rate of the eight representative slices in the vena cava model.
(DOCX)
