# Supplementary material for: Study of helical flow inducers with different thread pitches and diameters in vena cava
Source: PLoS One. 2018 Jan 3;13(1):e0190609. doi: 10.1371/journal.pone.0190609 (PMC5752007; doi:10.1371/journal.pone.0190609)
Supplement: S1 Table — A-D. Original data of helicity and shear rate of the eight representative slices in the vena cava model. (DOCX) [file pone.0190609.s001.docx]

**Supporting Information**

**S1 Table A-D.** Original data of Helicity and shear rate of the eight representative slices in the vena cava model.

**Table A. Helicity of eight slices (Carreau-model).**

| Slice | A | B | C | D | **E** |
| --- | --- | --- | --- | --- | --- |
| S1 | 0.15021 | 0.13985 | 0.12978 | 0.11373 | 0.11636 |
| S2 | 0.24444 | 0.15419 | 0.14772 | -0.0289 | 0.09782 |
| S3 | 0.20708 | 0.18801 | 0.17204 | -0.04955 | 0.10309 |
| S4 | 0.16697 | 0.13611 | 0.16677 | -0.02072 | 0.06616 |
| S5 | 0.1184 | 0.09451 | 0.09445 | 0.00985 | 0.03915 |
| S6 | 0.09495 | 0.06647 | 0.05443 | 0.00741 | -0.0047 |
| S7 | 0.05933 | 0.05147 | 0.03631 | 0.00785 | -0.01077 |
| S8 | 0.04929 | 0.04186 | 0.03478 | 0.01013 | -0.00756 |

**Table B. Helicity of eight slices (Newton-model).**

| Slice | A | B | C | D | E |
| --- | --- | --- | --- | --- | --- |
| S1 | 0.21214 | 0.19785 | 0.18343 | 0.17055 | 0.167 |
| S2 | 0.24013 | 0.17547 | 0.17223 | 0.02064 | 0.1471 |
| S3 | 0.22802 | 0.20729 | 0.20506 | -0.01318 | 0.15128 |
| S4 | 0.17962 | 0.14517 | 0.19911 | -0.0087 | 0.09632 |
| S5 | 0.13633 | 0.09688 | 0.10375 | 0.01129 | 0.05859 |
| S6 | 0.11688 | 0.0498 | 0.0444 | -0.00582 | -0.00733 |
| S7 | 0.07908 | 0.04342 | 0.03391 | 0.00702 | -0.00491 |
| S8 | -0.07027 | 0.0443 | 0.04062 | 0.02183 | 0.00558 |

**Table C. Shear rate of eight slices (Carreau-model).**

| Slice | A | B | C | D | E |
| --- | --- | --- | --- | --- | --- |
| S1 | 40.04592 | 41.71097 | 37.26028 | 41.17183 | 34.23866 |
| S2 | 54.01631 | 56.44205 | 35.39031 | 45.74365 | 30.8768 |
| S3 | 46.07836 | 44.04337 | 31.70938 | 39.17266 | 28.11627 |
| S4 | 28.78429 | 27.36122 | 26.0835 | 28.83587 | 21.36205 |
| S5 | 18.43431 | 17.30607 | 18.9034 | 20.76147 | 17.85485 |
| S6 | 15.53692 | 13.53395 | 15.77742 | 15.13792 | 16.16742 |
| S7 | 12.72053 | 11.100002 | 13.06629 | 11.65435 | 13.51938 |
| S8 | 12.16456 | 10.5298 | 12.46942 | 10.73028 | 12.80034 |

**Table D. Shear rate of eight slices (Newton-model).**

| Slice | A | B | C | D | E |
| --- | --- | --- | --- | --- | --- |
| S1 | 41.10188 | 42.71768 | 38.53076 | 42.69383 | 35.39828 |
| S2 | 58.03434 | 60.44414 | 37.89976 | 47.7552 | 32.25193 |
| S3 | 50.42624 | 47.4829 | 34.91996 | 41.37767 | 30.28562 |
| S4 | 32.37335 | 30.34768 | 29.41961 | 31.57039 | 24.00598 |
| S5 | 20.49491 | 20.23373 | 20.93838 | 24.62603 | 21.08809 |
| S6 | 16.32522 | 15.47529 | 17.95688 | 19.52178 | 19.89581 |
| S7 | 13.52671 | 12.77395 | 15.18345 | 15.55145 | 17.51368 |
| S8 | 13.21981 | 12.13096 | 14.86655 | 14.43695 | 16.68871 |
